# Sanguinary Calculus?

**Published:** 1882-09

**Authors:** 


### EDITORIAL.

### "SANGUINARY CALCULUS?"

The roots of teeth involved in abscess often have upon them calcarious deposits, these are sometimes found in considerable quantity, but usually in the form of small granules, which may be located at any point upon the surface of the roots. This deposit differs from the ordinary salivary calculus. It is not deposited from the saliva, it is uniformly more dense than salivary calculus, it is darker and never accumulates in as large quantities. The question of its origin has been one of doubt and uncertainty in the minds of many, at least. With the present comprehension of diseases of the teeth, and especially of alveolar abscess and the various phases that attend it, the occurence of this deposit is no longer a matter of mystery or uncertainty. The calcarious material is, previous to its deposition, held in solution by the fluid, pus or sanies, that surrounds the root in an abscess, and the deposition is made from this just as from any other menstruum. The blood is no more a factor in causing the presence of this material in this menstruum and its precipitation upon the roots, than in causing its presence in the saliva and its precipitation upon the necks and crowns of the teeth. The saliva is separated from the blood with its calcarious material in solution. The purulent or sanious matter is in part the degenerated or decomposed plasm wept or exuded through the walls of the capillaries in the diseased territory, and in part the debris of the decomposing tissue of both the hard and soft structures involved in the destructive process. Now, from the fluid mass thus produced, this deposit upon the roots of the teeth is made, and it is precipitated just as any material is precipitated from its solvent, when it is no longer able to hold it in solution. But why is it precipitated upon the roots of the teeth? why is it not washed out with the discharge of fluid? Perhaps a very large proportion of it is thus disposed of, but that which is precipitated near to or in contact with the roots of the teeth, there find points of lodgment and becomes attached; just as calculus in saliva. The precipitation, deposit and accretion of urinary calculus is far more occult and mysterious than this sanious calculus upon the roots of teeth having old abscesses. In the bladder, the first precipitation that finds lodgment is not voided, and is a nucleus upon which subsequent precipitation takes place, and in proportion as the mass grows does it offer greater surface and facility for deposition.

The term *sanguinary calculus* is no more applicable to this root deposit than to any other precipitated calculi in the body.

It is calculated to lead estray and to give a false impression. Suppose we call it *sanious calculus.* How will that do brother Ingersoll? If you have a better name, bring it on and we will adopt it, but let us drop the term *sanguinary calculus,* and find some name that will not do violence to truth and science.

